# Integrated impact of climate change on health outcomes and economic stability in PEPFAR-supported African countries

**DOI:** 10.1093/haschl/qxag050

**Published:** 2026-04-28

**Authors:** Sachin Silva, Veronique Whittaker, Eric Goosby, Michael J A Reid

**Affiliations:** Center for Global Health Delivery, Diplomacy, and Economics, Institute for Global Health Sciences, University of California, San Francisco, San Francisco, CA 94158, United States; Center for Global Health Delivery, Diplomacy, and Economics, Institute for Global Health Sciences, University of California, San Francisco, San Francisco, CA 94158, United States; Center for Global Health Delivery, Diplomacy, and Economics, Institute for Global Health Sciences, University of California, San Francisco, San Francisco, CA 94158, United States; School of Medicine, University of California, San Francisco, San Francisco, CA 94110, United States; Center for Global Health Delivery, Diplomacy, and Economics, Institute for Global Health Sciences, University of California, San Francisco, San Francisco, CA 94158, United States; School of Medicine, University of California, San Francisco, San Francisco, CA 94110, United States

**Keywords:** climate change, full income losses, sub-Saharan Africa, PEPFAR

## Abstract

**Introduction:**

Health impacts of global warming in sub-Saharan African countries that received President's Emergency Plan for AIDS Relief (PEPFAR)–funded HIV support are not known.

**Methods:**

Assuming the narrative of the Shared Socioeconomic Pathway 2, we estimated excess deaths, life expectancy losses at birth, and economic welfare losses in terms of full income. We relied on the MAGICC climate model for temperature predictions from 2025–2100 and net all-cause mortality risks estimated by others.

**Results:**

Surface temperature increases could reduce life expectancy at birth by 0.057 years in 2025 (95% CI: 0.024–0.095). By 2050, the reduction could increase by 30.7%, to 0.075 years (95% CI: 0.0311–0.1247). By 2100, it could further increase by 44.5%, to 0.083 years (95% CI: 0.034–0.138). Corresponding full income losses are US$11.44 billion in 2025 (95% CI: $4.77–$19.07 billion), which increases by 4-fold in 2050 to US$44.62 billion (95% CI: $18.65–$74.36 billion). By 2100, a 30-fold increase is possible, to US$353.56 billion (95% CI: $148.84–$588.32 billion). On a per capita basis, the highest full income losses consistently accrue to Lesotho between 2025 and 2100 (US$20.51, or 0.70% of per capita GDP, to US$355.39, or 0.80%).

**Conclusion:**

Adjusted investment is needed to address climate impacts, especially in countries such as Lesotho that may bear damage due to other regional emitters.

## Introduction

The evolving climate crisis has unmaskedfrailties in health systems. African countries are especially vulnerable because of lower adaptive capacity due to modest economic diversification and high dependence on agroecosystems.^1^ For high HIV-burden countries in Africa, the climate crisis is a threat-multiplier^2^ that has the potential to undermine hard-won progress.^3^ In November 2024, the UN Climate Change Conference (COP29) reached a New Collective Quantified Goal on Climate Finance (NCQG), pledging to increase financing to developing countries from US$100 billion to US$300 billion by 2035. The NCQG fell far short^4^ of the US$1.3 trillion needed globally.^4^ COP30, held in November 2025, did little to mitigate the shortfalls. The final agreement, which included a pledge to triple adaptation funding, although a positive signal, delays funding to 2035 from 2030.^5^ These new fiscal uncertainties underscore the importance of strengthening domestic health systems and increasing resilience to climate-related health threats.

Climate events, such as rainfall, flooding, and droughts, are thought to fuel HIV transmission^6,7^ and noncontinuity of HIV care^8^ via 3 pathways: climate events disrupt agricultural output and livelihoods leading to food insecurity and economic vulnerability, which increase the likelihood of transactional sex as individuals seek alternative income and food sources^9,10^; climate events also increase the likelihood of population migration, which can increase high-risk partnerships. Among climate events, heat and droughts have exacted the heaviest toll in Africa, with 64% of land area affected by at least 1 month of extreme drought per year. Africa also saw the highest loss in income due to heat stress in 2022, wiping out the equivalent of 4.1% of GDP (Gross Domestic Product), mainly from losses in the agricultural sector.^11^ While adaptation measures, such as improved housing and strengthened public health systems, have dramatically reduced heat-related health risks in other regions,^12,13^ in Africa, due to the compounding of the burden of HIV with that of tuberculosis^14^ and malaria,^15^ both of which are known to be associated with climate events,^16,17^ they have had a marginal effect.^18^

To our knowledge, the relationship between temperature, HIV prevalence, and all-cause mortality have received limited quantitative treatment in the academic literature. The studies by Carleton et al^19^ and by Baker^20^ remain some of the few studies quantifying the impact of surface temperature increases on HIV prevalence. Understanding the full economic consequences is imperative if climate finance is to adequately account for losses inclusive of health effects. Such an assessment is all the more important given that most climate models from which social cost of carbon (SCC) estimates are derived minimally account for health-related losses.^21^ Even those that do, such as the Climate Framework for Uncertainty, Negotiation and Distributionmodel, while accounting for morbidity and mortality,^22^ do not account for the economic value derived from living longer and healthier lives.

There is a particular paucity of data describing the health effects of global warming in sub-Saharan African (SSA) countries that received President's Emergency Plan for AIDS Relief (PEPFAR)–funded HIV support, especially estimates of economic welfare costs inclusive of the added value of living longer and productive lives. In this analysis, we sought to quantify these losses adopting a full-income approach,^23-25^ as well as contributing deaths and life expectancy losses. We focus on deaths directly attributable to surface temperature changes due to the availability of reliable data, acknowledging that other climate-related health impacts, such as vector-borne diseases and waterborne illnesses, may also be influenced by climate change. Our methodology aims to link all-cause mortality attributable to temperature-related health effects within high-HIV-burden countries, thereby providing a clearer context for understanding the impacts on their health systems.

## Data and methods

To approximate the current climate trajectory, we assumed that countries would follow the socioeconomic narrative consistent with the Shared Socioeconomic Pathway 2 (SSP2)^26^. We extracted corresponding temperature increases from preindustrial levels (1850–1900), projected for 2025 to 2100 by the Model for the Assessment of Greenhouse Gas Induced Climate Change (MAGICC) version 7.0, a prime reduced-complexity model often used by a number of integrated assessment models.^27,28^ For reported annual temperatures, we calculated net all-cause mortality risks using the net change in all-cause mortality risk for a unit change in temperature estimated by Cromar et al.^29^ We applied these risks to age-specific annual mortalities from 2025 to 2100 in 19 SSA countries, which we derived based on population projections for the SSP2 scenario by Samir et al,^32,33^ and mortality projections from the UN World Population Prospects (WPP).^34^ The countries included Angola, Botswana, Burundi, Cote d'Ivoire, Cameroon, Democratic Republic of Congo, Eswatini, Ethiopia, Kenya, Lesotho, Malawi, Mozambique, Namibia, Nigeria, Rwanda, South Africa, United Republic of Tanzania, Zambia, and Zimbabwe, all of which are recipients of HIV-specific donor financing, as part of the US government's PEPFAR initiative.^35^ Assuming that net all-cause mortality risks remained invariant by age and sex, we estimated median life expectancy losses at birth (LE0) due to the excess deaths by constructing multi-decrement life tables^36^ for each country by year, eliminating excess deaths and thereby eliminating health effects of surface temperature increases as a contributor to all-cause mortality.

We valued the contribution of excess deaths towards full-income losses by first transforming the excess hazard of mortality to standardized mortality units (a 1 in 10 000 change in mortality risk), re-scaling it to age 35 years, calculating its population value by country and year and then multiplying by the per capita GDP for the SSP2 scenario projected by Dellink et al^37^ and the value of a standardized mortality unit calculated as a proportion of the per capita GDP.^25^ To calculate the standardized mortality unit, we first calculated the value of a statistical life (VSL) using benefits transfer and US VSL for 2024, assuming an income elasticity of 1.0.^38^ We re-scaled to age 35 as most VSL literature assesses the value of reducing mortality at middle age.^25^ We report all values in 2024 US$ rates. We evaluated sensitivity to parameter uncertainty and choice using a Latin hypercube sampling algorithm^39^ (Supplementary Appendix Methods, Figure S1, Table S1).

## Results

In the 19 countries that we analyzed, the SSP2 scenario predicts 6 890 077 deaths in 2025, 9 593 426 deaths in 2050, and 20 753 502 deaths in 2100.^32,40^ In 2025, 0.33% of these deaths would be due to the health effects of surface temperature increases. In 2050, the share increases to 0.33%, and in 2100 to 0.48%. The deaths in 2025 would contribute to a reduction in life expectancy losses at birth of 0.057 years (95% CI: 0.024–0.095 years); in 2050, to 0.075 years (95% CI: 0.031–0.125 years); and in 2100, to 0.083 years (95% CI: 0.034–0.138 years). In 2025, the highest life expectancy losses at birthwould be in Nigeria (0.07 years; 95% CI: 0.029–0.116), followed by Zimbabwe (0.069 years; 95% CI: 0.029–0.115) and Lesotho (0.07 years; 95% CI: 0.029–0.115). In 2100, they would also be in Lesotho (0.099 years; 95% CI: 0.042–0.165) and Nigeria (0.097 years; 95% CI: 0.040–0.163), followed by Zimbabwe (0.091 years; 95% CI: 0.038–0.152) (Table 1).

**Table 1.** Total deaths, median life expectancy losses, and total full-income losses due to health effects of global warming in 19 highest-HIV-burden countries in sub-Saharan Africa currently receiving PEPFAR funding—2025 to 2100 in 5-year increments.

Year	Total deaths	No. of deaths due to health effects of global warming (95% CI)	Median life expectancy losses at birth, y (95% CI)	Total full-income losses (in 2022 US$ billions) (95% CI)
2025	6 890 077	22 716 (9465-37 861)	0.0571 (0.0242-0.0955)	11.44 (4.77-19.07)
2030	7 310 118	25 839 (10 766-43 065)	0.0623 (0.0260-0.1039)	15.07 (6.31-25.08)
2035	7 781 180	29 980 (12 492-49 967)	0.0669 (0.0275-0.1120)	19.89 (8.32-33.14)
2040	8 302 470	34 537 (14 390-57 562)	0.0685 (0.0285-0.1144)	26.1 (10.88-43.51)
2045	8 908 653	39 471 (16 446-65 786)	0.0717 (0.0299-0.1197)	34.08 (14.26-56.77)
2050	9 593 426	45 557 (18 982-75 929)	0.0746 (0.0311-0.1247)	44.62 (18.65-74.36)
2055	10 383 877	51 484 (21 451-85 806)	0.0792 (0.0326-0.1327)	57.21 (23.92-95.34)
2060	11 230 517	58 457 (24 357-97 429)	0.0803 (0.0337-0.1336)	73.11 (30.56-121.87)
2065	12 170 076	65 629 (27 345-109 381)	0.0808 (0.0336-0.1348)	92.2 (38.55-153.67)
2070	13 203 568	74 431 (31 013-124 051)	0.0855 (0.0356-0.1429)	116.95 (48.96-194.87)
2075	14 294 220	82 940 (34 558-138 233)	0.0819 (0.0344-0.1368)	144.63 (60.55-241.01)
2080	15 459 179	92 520 (38 550-154 200)	0.0856 (0.0357-0.1428)	178.48 (74.82-297.33)
2085	16 663 547	102 159 (42 566-170 265)	0.0861 (0.0359-0.1437)	215.7 (90.39-359.38)
2090	17 962 998	112 128 (46 720-186 880)	0.0839 (0.0349-0.1399)	256.71 (107.55-427.74)
2095	19 320 956	122 855 (51 190-204 759)	0.0841 (0.0352-0.1400)	303.85 (127.43-506.15)
2100	20 753 502	132 887 (55 370-221 479)	0.0825 (0.0344-0.1381)	353.56 (148.84-588.32)

Total deaths are reported for ages 0–95 years. Median life expectancy losses at birth represent gains if mortality due to health effects of global warming were eliminated from all-cause mortality for each year. Full-income losses were calculated on the basis of GDP per capita for each country and year predicted by population and GDP projections for SSP2 by Samir et al.^32,33^ and Dellink et al,^37^ respectively. The upper and lower bounds represent deaths, life expectancy losses, and full-income losses corresponding to the upper and lower bounds of the net change in all-cause mortality risk for a unit change (1°C) in surface temperature for sub-Saharan Africa reported by Cromar and colleagues.^29^ All values reported are single-year estimates for the respective years.

Abbreviations: GDP, Gross Domestic Product; PEPFAR, President's Emergency Plan for AIDS Relief; SSP2, Shared Socioeconomic Pathway 2.

The 22 716 deaths in 2025 and the resulting 0.057 years in life expectancy losses at birth equate to US$11.44 billion in full-income losses (95% CI: $2.97–$11.87 billion). Nearly 40% of these losses (39.88%; US$4.56 billion) are in Nigeria, which accrues the highest number of deaths (8873) and thehighest life expectancy losses at birth (0.069 years). Full-income losses as a share of GDP range between 1.12% (US$4.56 billion in Nigeria) and 0.4% (US$61.33 millionin Rwanda). As a share of per capita GDP, they range between 0.7% (US$20.51) and 0.25% (US$4.03) in the same countries. On a per-person basis however, the losses range between US$52.29 (in Botswana) and US$3.41 (in Burundi) (Tables 1 and 2; Table S2).

**Table 2.** Total deaths, median life expectancy losses, and total full-income losses due to health effects of global warming (by country) for the 19 highest-HIV-burden countries in sub-Saharan Africa currently receiving PEPFAR funding—2025 to 2100.

Country	Total deaths	No. of deaths due to health effects of global warming (95% CI)	Median life expectancy losses at birth, y (95% CI)	Total full-income losses (in 2022 US$ billions) (95% CI)
Angola	6 767 738	37 417 (15 590-62 361)	0.0783 (0.0323-0.1309)	64.59 (26.88-107.81)
Burundi	3 001 511	16 437 (6849-27 395)	0.0782 (0.0322-0.1309)	15.49 (6.45-25.84)
Botswana	489 406	2647 (1103-4411)	0.0782 (0.0326-0.1304)	7.25 (3.02-12.11)
Cote d'lvoire	6 224 172	33 811 (14 088-56 352)	0.0885 (0.0366-0.1480)	76.86 (31.98-128.33)
Cameroon	5 945 712	32 394 (13 497-53 990)	0.0843 (0.0354-0.1403)	49.65 (20,67-82.89)
Democratic Republic of the Congo	23 018 602	126 293 (52 622-210 488)	0.0834 (0.0349-0.1389)	223.93 (93.31-373.66)
Ethiopia	20 169 848	110 037 (45 849-183 395)	0.0663 (0.0272-0.1110)	114.48 (47.63-191.10)
Kenya	11 866 863	64 890 (27 037-108 150)	0.0772 (0.0318-0.1292)	125.28 (52.12-209.15)
Lesotho	582 467	3085 (1285-5141)	0.0974 (0.0409-0.1623)	5.03 (2.11-8.39)
Mozambique	6 695 178	36 471 (15 196-60 785)	0.0698 (0.0288-0.1168)	37.99 (15.84-63.39)
Malawi	4 808 440	26 719 (11 133-44 531)	0.0752 (0.0313-0.1255)	32.74 (13.62-54.66)
Namibia	671 188	3638 (1516-6064)	0.0811 (0.0339-0.1352)	8.26 (3.431-13.83)
Nigeria	78 536 536	429 318 (178 883-715 531)	0.0945 (0.0392-0.1576)	871.73 (368.55-1488.38)
Rwanda	2 697 730	14 781 (6159-24 635)	0.0709 (0.0295-0.1185)	14.09 (5.86-23.52)
Eswatini	240 521	1284 (535-2139)	0.0833 (0.0345-0.1393)	1.96 (0.84-3.25)
United Republic of Tanzania	11 484 077	63 340 (26 391-105 566)	0.0689 (0.0287-0.1149)	68.10 (28.33-113.67)
South Africa	10 610 524	56 031 (23 346-93 385)	0.0809 (0.0337-0.1349)	161.92 (67.36-270.35)
Zambia	3 925 644	21 642 (9018-36 071)	0.0804 (0.0327-0.1344)	33.87 (14.10-56.53)
Zimbabwe	2 492 207	13 358 (5566-22 262)	0.0880 (0.0366-0.1467)	30.40 (12.64-50.76)

Total deaths are reported for ages 0–95 years. Full-income losses were calculated on the basis of GDP per capita for each country and year calculated based on population and GDP projections for SSP2 by Samir et al^32,33^ and Dellink et al,^37^ respectively. The upper and lower bounds represent deaths, life expectancy losses, and full-income losses corresponding to the upper and lower bounds of the net change in all-cause mortality risk for a unit change (1°C) in surface temperature for sub-Saharan Africa reported by Cromar and colleagues.^29^ All values reported are single-year estimates for the respective years.

Abbreviations: GDP, Gross Domestic Product; PEPFAR, President's Emergency Plan for AIDS Relief; SSP2, Shared Socioeconomic Pathway 2.

By 2050, these full-income losses quadrupole, to US$44.62 billion (95% CI: $11.61–$46.30 billion), with Nigeria's contribution increasing to 44.24% (US$19.74 billion, due to 17 899 deaths and 0.088 years in life expectancy losses at birth). As a share of per capita GDP, losses range between 0.72% (US$53.50 million and US$45.89 billion in Nigeria and Lesotho, respectively) and 0.24% (US$12.99 billion in Tanzania). On a per-person basis, the losses range from US$109.31 (in Botswana) to US$10.41 (in Malawi) (Tables 1 and 2; Table S3).

By 2100, full-income losses increase by more than 30-fold from 2025, to US$353.56 billion (95% CI: $92.67–$366.28 billion). Nigeria contributes to nearly half of these losses (US$160.85 billion or 45.49%). As a share of per capita GDP, they range between 0.8% (US$355.29) in Lesotho, which surpasses Nigeria's 0.67% and 0.3% (US$91.28 billion) in Tanzania. On a per-person basis, they range from US$524.74 in Zimbabwe to US$87.31in Rwanda. On a per-capita GDP basis, the highest losses are consistently in Nigeria and Lesotho; on an absolute basis, they are consistently in Nigeria (Tables 1 and 2; Table S4).

### Sensitivity analysis

We found that full income is most sensitive to the income elasticity assumed. When allowed to vary between 0.5 and 1.5,^41^ full income ranged from US$1.40 and 42.30 billion in 2025. It was least sensitive to the US VSL value assumed (varied between US$3.32 and 10.84 billion in 2025 when the US VSL varied between $5 992 367 and $19 546 532^41^). The sensitivity to net all-cause mortality risk, which we allowed to vary between 1.001 and 1.004,^29^ was US$2.96 to 11.87 billion (in 2025). By 2100, however, full income becomes most sensitive to net all-cause mortality risk (varies between US$92.66 and 366.28 billion) followed by income elasticity (varies between US$128.77 and 379.27 billion) and US VSL assumed (varies between US$102.72 and 335.08 billion) (Tables S5 and S6; Figures S2–S4).

## Discussion

In the 19 countries that we analyzed, deaths due to health effects of rising surface temperatures will increase by nearly 6-fold from 2025 to 2100. The resulting life expectancy losses at birth will increase by nearly 1.5-fold, with full-income losses increasing by a staggering 30-fold. In 2025 and 2050, more than 40% of full-income losses will accrue to Nigeria. Indeed, Nigeria and South Africa accrue more than twice the full-income losses of all other countries combined. This finding is consequential because Nigeria and South Africa account for the highest AIDS-related deaths in adults (≥15 years).^42^ Nigeria and South Africa are also responsible collectively for more than one-third of the global greenhouse gas emissions (GHGEs) from the region.^43^

On a per-capita basis, however, the highest full-income losses consistently accrue to Lesotho, in addition to Nigeria. By 2100, full-income losses in Lesotho, as a share of per capita GDP, will exceed Nigeria's (0.8% in Lesotho compared with 0.67% in Nigeria). Although Lesotho's GHGEs in absolute terms is a fraction of Nigeria's, on a per-capita basis (in tonnes of CO_2_ equivalent), they exceed Nigeria's by nearly 30%.^43^ In contrast, Lesotho's GHGEs are less than an eighth of the per-capita GHGEs of South Africa (2013–2017), which is the highest emitter in the region, even exceeding China. However, per-capita full-income losses in South Africa are less than half of Lesotho's.^43^ This suggests that countries such as Lesotho bear the brunt of the damages due to dominant regional emitters such as South Africa. Mostefaoui et al^43^ also found that Lesotho is the third highest emitter when considering emissions per a unit of per-capita GDP gained (in purchasing power parityUS$ in 2016), next to Libya and South Africa. Lesotho is thus also vulnerable to losses due to its own actions as well as because of an emission-intensive economy. Both these points also underscore the potential role of full income as a measure of climate injustice.^44^

Plausible drivers of these losses are population size, population age structure, and per-capita income. In 2025, the population of Nigeria was nearly twice the population of the next most-populouscountry (Ethiopia). By 2100, it will be nearly 3 times. Yet, population projections for SSP2 suggest that, until 2040, the working-age population will not surpass the dependency population, assuming that recent declines in fertility rates continue. In contrast, Ethiopia is projected to experience a more favorable age structure, with a growing proportion of its population entering the working-age bracket.^32,33^ This difference makes population age structure a less significant factor in Nigeria's projected losses compared with Ethiopia's, where a more balanced age distribution could potentially mitigate the impact of rising mortality. Considering GDP, Nigeria's predicted per-capita GDP in 2025 is 1.3 times higher than that of the next country, but by 2100, these figures converge,^37^ indicating that economic disparities may diminish over time. This equilibration suggests that population size, rather than per-capita income alone, is the more likely driver of projected deaths and full-income losses. If the socioeconomic pathways shift—transitioning from SSP2 to either SSP1 or SSP3—the impact on absolute deaths and income losses could be substantial. Furthermore, if the all-cause mortality risk remained invariant, absolute deaths and full-income losses could vary substantially from estimated values because, during the second half of the century, SSP3 would give rise to a 12.6 billion global population in 2100, whereas SSP1 would result in a reduction to 6.9 billion, which is lower than today's population.^32,33^ Understanding these dynamics is crucial for anticipating the health and economic implications of climate change.

The driver not represented in our analysis is predicted HIV incidence. Established estimates of HIV incidence over the time scales considered are lacking. However, in South Africa, where longer-term projections are available, incidence is predicted to steadily decline from 2025 (from 4.95 per 1000 population; 95% CI: 4.40–5.34) to 2100 (to 0.14; 95% CI: 0.05–0.31).^45^ If future HIV incidence is expected to decline but deaths due to health effects of global warming are expected to rise, then either those deaths are rising due to other causes or global warming is contributing to a larger share of HIV deaths. For example, for South Africa, we estimated 1593 deaths in 2025 and 1811 deaths in 2030 (a 13.7% increase). Rautenbach et al^45^ estimated 46 192 HIV deaths in 2025 and 40 849 deaths in 2030. This 11.6% decline is potentially offset by the 13.7% increase in deaths due to rising temperatures.

In either case, our analysis suggests that donor assistance for HIV programs in SSA should take into account climate modifiers of infectious disease incidence trajectories,^46^ especially tuberculosis, and Malaria whose incidence is known to be associated with rising temperatures.^16,17^ Importantly, these findings arise at a moment when PEPFAR-supported countries are navigating declining external financing and expanded expectations for domestic ownership. In recent months, PEPFAR has entered into a Memoranda of Understanding with partner governments in SSA, aligning reductions in US support with projected increases in domestic resource mobilization for HIV. Countries such as Ethiopia and Lesotho are also well positioned to take advantage of a future demographic dividend,^47^ which can yield economic growth,^48^ making it possible to increase their share of co-financing. If realized, these domestic investments create an opportunity to strengthen health systems in ways that both sustain HIV gains and improve resilience to climate shocks. However, our projections suggest that climate-attributable mortality and associated welfare losses can erode fiscal space and increase health system strain during this transition. The interaction between climate stress and contracting donor support underscores the need to integrate climate resilience explicitly into HIV financing strategies, service delivery models, and long-term sustainability planning.

Among the limitations of our study, the reliance on estimates of net all-cause mortality risks estimated by Cromar et al^29^ is paramount. Two studies from Africa^30,31^ contributed to these estimates, neither of which accounted for the effects or costs of adaptation. Similarly, the net all-cause mortality estimates were not country-specific, which may obscure variations in health outcomes that could affect resource allocation and intervention strategies in individual countries. We had also assumed that this risk is invariant by age and gender, which is an additional limitation. In the same vein, we had assumed that the global mean changes in surface temperatures predicted by the MAGICC model^27,28^ are a reasonable approximation of local temperatures. Our estimates only account for all-cause mortality risks due to rising temperatures and not other climate events. Nor do our estimates account for changes in HIV mortality that may occur if climate shocks undermine health system capacity. The use of the US VSL to estimate country-specific VSLs is also a limitation. To assess the extent to which full-income estimates would vary should a country-specific VSL be used, we re-estimated full-income losses using empirically established values for Tanzania,^49^ adjusting for country income per capita. Last, the time scales considered in the analysis introduce uncertainty to parameter values, especially the life tables provided by the WPP.^34^

By quantifying the economic losses associated with climate-related health effects, we highlight the critical importance of integrating climate adaptation strategies into HIV programs. Through our findings, it becomes evident that climate change acts as an importantmodifier of health trajectories in HIV-endemic regions, necessitating integrated approaches to funding and program design. We urge stakeholders to consider the dual impact of climate-related health effects and HIV burden when formulating policies and resource allocations. Future funding and policy decisions must consider the interplay between climate change and infectious disease trajectories to ensure sustainable progress against HIV/AIDS and other major health threats in the region.

## Contribution statement

S.S. conceived the study, designed and conducted the analysis, and wrote the initial draft of the manuscript and led finalization of the manuscript. E.G. and V.W. reviewed and contributed to the manuscript. M.J.A.R. conceived the study (with S.S.) and wrote the initial draft with S.S. and contributed to finalizing the manuscript. All authors contributed to the discussion and interpretation of the results and to the final draft of the manuscript. All authors have read and approved the final manuscript.

## Supplementary Material

qxag050_Supplementary_Data

## Data Availability

Data generated or analyzed during this study are included in the main text and the supplementary material. The model code programmed in STATA can be obtained by contacting the authors.
