# Supplementary material for: Integrated impact of climate change on health outcomes and economic stability in PEPFAR-supported African countries
Source: Health Aff Sch. 2026 Apr 28;4(4):qxag050. doi: 10.1093/haschl/qxag050 (PMC13122613; doi:10.1093/haschl/qxag050)
Supplement: qxag050_Supplementary_Data [file qxag050_supplementary_data.zip › Appendix_Clean (2).docx]

**Integrated Impact of Climate Change on Health Outcomes and Economic Stability in High HIV-Burden Countries**

**Supplementary Appendix**

Sachin SILVA^1^, Veronique WHITTAKER^1^, Eric GOOSBY^1, 2^, Michael JA REID^1,2^

1. University of California, San Francisco

Center for Global Health Delivery, Diplomacy and Economics

Institute for Global Health Sciences

550 16th Street, Third Floor

San Francisco, CA 94158 USA

1. University of California, San Francisco

School of Medicine

1001 Potrero Avenue, #3777

San Francisco, CA 94110 USA

**Corresponding author:**

Sachin Silva DrPH

University of California, San Francisco

Center for Global Health Delivery, Diplomacy and Economics

Institute for Global Health Sciences

550 16th Street, Third Floor

San Francisco, CA 94158 USA

Email: sas7443@mail.harvard.edu

**Methods**

*Reference case*

We took a societal perspective and estimated costs accrued to individuals/households and the government. We estimated economic costs rather than the financial costs – meaning the value of the resources as an opportunity cost, rather than the value of payments. Our estimates are therefore independent of payment structures. We also estimate full costs rather than incremental costs. For the 19 countries, we estimated costs annually from 2025 to 2100 in five-year increments and report them in 2022 US$ rates.

*Estimating approach*

We extracted temperature increases from pre-industrial levels (1850-1900), projected for 2025 to 2100 for the Shared Socioeconomic Pathway 2 (SSP2) by the Model for the Assessment of Greenhouse Gas Induced Climate Change (MAGICC) version 7.0^1,2^. For each annual temperature increase, we calculated the corresponding net all-cause mortality risks using the net change in all-cause mortality risk for a unit change in temperature estimated by Cromar et al. ^5^. We applied these risks to age-specific annual mortalities from 2025 to 2100 in 19 SSA countries, which we derived based on population projections for the SSP2 scenario by Samir et al.^3,7^ , and mortality rates from the UN World Population Prospects (WPP)^8^.

*Life expectancy calculation*

We used median variant population[ref] and mortality projections from the UN World Population Prospects (WPP)^9^ to calculate an annual crude mortality rate for each age interval matching the age intervals in the abridged lifetables provided by WPP. We applied this crude mortality rate to population projections for SSP2 provided by Samir et al^3,7^ to calculate the expected deaths for SSP2 from 2025 to 2100 (in 5-year increments). To estimate the share of deaths due to health effects of surface temperature increases, we first calculated annual all-cause mortality risks using temperature projections from the MAGICC7.0 model^1,2^ and estimates of the net change in all-cause mortality risk for a unit change (1^0^C) in ambient temperature for sub-Saharan Africa by Cromar and colleagues^5^. Using these deaths and the total deaths, we calculated life expectancies at birth from 2025 to 2100 (in 5-year increments) using standard multi-decrement life table methods^10^.

*Full income calculation*

We first transformed the excess hazard of mortality (due to surface temperature increases) to standardized mortality units (a 1 in 10 000 change in mortality risk) which we then rescaled to life expectancy at age 35. The rescaling permitted us to transform the value of the risk change to a value of annual income, based on empirical estimates linking mortality risk change to life expectancy change and annual income changes, which use age 35 as the reference age.

$$\Delta smu\left( a \right)=\frac{e\left( a \right)}{e\left( 35 \right)}\Delta smu\left( e_{0}, e_{TB} \right){n(a)}$$

We then calculated the population value of this risk change and aggregated the value for each country and year. We multiplied this value by the value of a standardized mortality unit (VSMU) which we calculated as a proportion of the predicted income per capita. We then multiplied by the predicted per capita income for SSP2 from Dellink et al^11^. In calculating the VSMU, we used the country value-of-a-statistical-life-year (VSLY), which we calculated using benefits transfer with the US VSL which we updated to 2022 from the value recommended by the U.S. Department of Health and Human Services (HHS) for 2013^12^ using their worksheet for updating for inflation and changes in real-income, and an income elasticity of 1.0^13^ (subjected to sensitivity analysis). In updating the VSL, we assumed 2020 US$ values and converted to 2022 US$ values.

$$V\left( e_{0}, e_{TB}, y \right)=y.\gamma\int_{0}^{\infty} n\left( a \right) \Delta smu\left( e_{0}, e_{TB} \right)\frac{e\left( a \right)}{e(35)}da$$

*
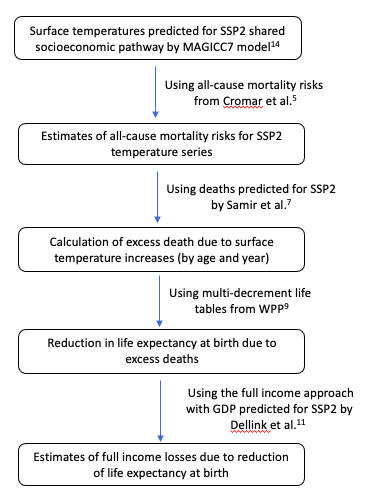
*

**Supplemental Figure 1: Estimating approach**

*Country selection*

We selected the twenty countries with the highest burden of HIV which were all recipients of HIV donor assistance from the US government through the President's Emergency Plan for AIDS Relief (PEPFAR) program. We excluded South Sudan as population projections for the SSP2 scenario were not available ^7^. The countries were also the most vulnerable to climate shocks per a recent assessment by the African Development Bank^14^

*Data Sources*

| ***Data*** | ***Source*** | ***Justification*** | ***Assumption*** |
| --- | --- | --- | --- |
| Temperatures corresponding to SSP2 from 2025-2100 | From MAGICC (version 7.0) prime reduced-complexity model^1,2^ | The model is often used by IPCC as well as by a number of Integrated Assessment Models. | Temperatures predicted are increases from pre-industrial levels (1850-1900) |
| Net all-cause mortality risk | From Cromar et al. ^5^ | Provides regionally resolved effect estimates of unit increases in temperature on net all-cause mortality taking into account cardiovascular,  respiratory, gastrointestinal, renal, endocrine, neurologic, psychiatric, obstetric, gynecologic, and infectious disease outcomes. | None |
| Annual populations corresponding to SSP2 scenario from 2025-2100 | From Samir et al.^7^ | The authoritative source for SSP related projections. Further confirmed via correspondence with the author. | None |
|  |  |  |  |
|  |  |  |  |
| Abridged life tables from 2025-2100 | From World Population Prospects (WPP)^9^ | To our knowledge, the most reliable source for lifetable projections from 2025-2100 for the countries of interest | None |
|  |  |  |  |
| Annual GDP per capita | From Samir et al.^7^ | The authoritative source for SSP related projections. Further confirmed via correspondence with the author. | GDP is reported in 2005 US$ rates. |
|  |  |  |  |

**Supplemental Table 1: Sources of data, justification for use, and assumptions or adjustments made when using**

**Data Tables**

| **Country** | **Deaths** | **Life expectancy losses (years)** | **Full income (FE) losses (US$)** | **Full income losses per person** | **Full income losses as a share of GDP per capita** | **Full income losses as a share of annual GDP** |
| --- | --- | --- | --- | --- | --- | --- |
| Angola | 641 | 0.05649 | $869,490,496 | $30.84 | 0.38% | 0.61% |
| Botswana | 61 | 0.05328 | $122,777,096 | $52.29 | 0.30% | 0.48% |
| Burundi | 302 | 0.06083 | $41,056,344 | $3.41 | 0.38% | 0.61% |
| CÃ´ted'Ivoire | 770 | 0.06512 | $352,736,096 | $14.31 | 0.46% | 0.74% |
| Cameroon | 681 | 0.05708 | $295,672,992 | $11.62 | 0.40% | 0.64% |
| DRC | 2,510 | 0.06139 | $491,318,400 | $5.18 | 0.48% | 0.77% |
| Eswatini | 37 | 0.06361 | $20,194,102 | $14.63 | 0.39% | 0.63% |
| Ethiopia | 2,313 | 0.05082 | $508,875,488 | $4.51 | 0.29% | 0.46% |
| Kenya | 1,261 | 0.05673 | $633,003,584 | $11.43 | 0.33% | 0.53% |
| Lesotho | 94 | 0.06886 | $31,294,136 | $12.77 | 0.61% | 0.98% |
| Malawi | 442 | 0.05452 | $78,502,856 | $3.48 | 0.29% | 0.47% |
| Mozambique | 823 | 0.05672 | $172,564,368 | $5.56 | 0.37% | 0.60% |
| Namibia | 79 | 0.06001 | $86,850,024 | $31.04 | 0.36% | 0.57% |
| Nigeria | 8,873 | 0.06953 | $4,560,072,192 | $20.51 | 0.70% | 1.12% |
| Rwanda | 277 | 0.04923 | $61,327,136 | $4.03 | 0.25% | 0.40% |
| South Africa | 1,593 | 0.05595 | $2,577,390,080 | $45.28 | 0.32% | 0.51% |
| Tanzania | 1,166 | 0.05028 | $303,439,648 | $4.67 | 0.26% | 0.41% |
| Zambia | 417 | 0.05877 | $136,908,384 | $7.29 | 0.36% | 0.59% |
| Zimbabwe | 377 | 0.06941 | $91,855,672 | $6.92 | 0.40% | 0.64% |

**Supplemental Table 2: Deaths, life expectancy losses, and full income losses as a share of annual GDP, as a share of per capita GDP (2025).** Full income losses per person were derived by dividing the full income losses by the annual population projected for SSP2 by Samir et al. ^197^. Full income losses as a share of GDP per capita was derived by dividing the full income losses per person by the per capita GDP projected for SSP2 by Dellink et al. ^19^. Full income losses as a share of annual GDP was derived by dividing the annual full income losses by the annual GDP projected for SSP2 by Dellink et al. ^19^. All monetary values are reported in 2024 US$ rates.

| **Country** | **Deaths** | **Life expectancy losses (years)** | **Full income (FE) losses**  **(US$)** | **Full income losses per person** | **Full income losses as a share of GDP per capita** | **Full income losses as a share of annual GDP** |
| --- | --- | --- | --- | --- | --- | --- |
| Angola | 1,434 | 0.077675 | $1,447,177,216 | $32.98 | 0.39% | 0.63% |
| Botswana | 115 | 0.078629 | $299,961,952 | $109.31 | 0.39% | 0.62% |
| Burundi | 688 | 0.072533 | $249,337,248 | $14.83 | 0.43% | 0.69% |
| CÃ´ted'Ivoire | 1,436 | 0.079224 | $2,005,324,800 | $65.43 | 0.52% | 0.84% |
| Cameroon | 1,378 | 0.082382 | $1,221,853,312 | $36.81 | 0.45% | 0.73% |
| DRC | 5,022 | 0.078621 | $3,653,627,648 | $25.17 | 0.47% | 0.76% |
| Eswatini | 59 | 0.074570 | $57,395,764 | $37.05 | 0.43% | 0.70% |
| Ethiopia | 4,611 | 0.062599 | $2,122,065,792 | $13.36 | 0.27% | 0.43% |
| Kenya | 2,736 | 0.072289 | $2,703,531,520 | $34.61 | 0.38% | 0.60% |
| Lesotho | 151 | 0.091320 | $122,668,272 | $45.89 | 0.72% | 1.15% |
| Malawi | 974 | 0.069908 | $420,482,944 | $10.41 | 0.30% | 0.48% |
| Mozambique | 1,487 | 0.068298 | $671,607,680 | $15.86 | 0.35% | 0.55% |
| Namibia | 161 | 0.079765 | $266,841,024 | $79.70 | 0.45% | 0.72% |
| Nigeria | 17,899 | 0.088951 | $19,740,850,176 | $53.50 | 0.72% | 1.15% |
| Rwanda | 623 | 0.061768 | $280,072,768 | $12.21 | 0.26% | 0.42% |
| South Africa | 2,918 | 0.074608 | $6,867,089,408 | $108.65 | 0.42% | 0.68% |
| Tanzania | 2,455 | 0.063148 | $1,327,144,704 | $12.99 | 0.24% | 0.39% |
| Zambia | 827 | 0.070450 | $656,526,976 | $21.89 | 0.37% | 0.59% |
| Zimbabwe | 583 | 0.077698 | $507,984,448 | $38.62 | 0.48% | 0.78% |

**Supplemental Table 3: Deaths, life expectancy losses, and full income losses as a share of annual GDP, as a share of per capita GDP (2050).** Full income losses per person were derived by dividing the full income losses by the annual population projected for SSP2 by Samir et al. ^197^. Full income losses as a share of GDP per capita was derived by dividing the full income losses per person by the per capita GDP projected for SSP2 by Dellink et al. ^19^. Full income losses as a share of annual GDP was derived by dividing the annual full income losses by the annual GDP projected for SSP2 by Dellink et al. ^19^. All monetary values are reported in 2024 US$ rates.

| **Country** | **Deaths** | **Life expectancy losses** | **Full income (FE) losses**  **(US$)** | **Full income losses per person** | **Full income losses as a share of GDP per capita** | **Full income losses as a share of annual GDP** |
| --- | --- | --- | --- | --- | --- | --- |
| Angola | 4,733 | 0.082321 | 11,909,955,584 | 208.78 | 0.48% | 0.77% |
| Botswana | 273 | 0.078514 | 791,556,288 | 286.02 | 0.49% | 0.79% |
| Burundi | 1,866 | 0.083374 | 3,156,435,968 | 166.51 | 0.52% | 0.84% |
| CÃ´ted'Ivoire | 3,888 | 0.091080 | 12,533,842,944 | 383.05 | 0.65% | 1.05% |
| Cameroon | 3,692 | 0.087158 | 8,489,676,288 | 235.77 | 0.56% | 0.91% |
| DRC | 15,747 | 0.088455 | 46,110,507,008 | 246.01 | 0.53% | 0.85% |
| Eswatini | 133 | 0.082451 | 329,684,672 | 217.02 | 0.58% | 0.93% |
| Ethiopia | 12,663 | 0.071129 | 22,381,756,416 | 116.94 | 0.32% | 0.51% |
| Kenya | 7,583 | 0.084610 | 22,911,350,784 | 239.44 | 0.46% | 0.73% |
| Lesotho | 296 | 0.099152 | 870,680,960 | 355.39 | 0.80% | 1.29% |
| Malawi | 3,567 | 0.081970 | 7,536,785,920 | 112.82 | 0.36% | 0.57% |
| Mozambique | 4,223 | 0.072769 | 7,801,637,888 | 155.21 | 0.41% | 0.65% |
| Namibia | 385 | 0.082458 | 891,915,072 | 269.36 | 0.56% | 0.89% |
| Nigeria | 54,890 | 0.097389 | 160,852,115,456 | 275.91 | 0.67% | 1.07% |
| Rwanda | 1,763 | 0.074234 | 2,702,306,816 | 87.31 | 0.32% | 0.51% |
| South Africa | 4,901 | 0.090576 | 18,416,750,592 | 314.49 | 0.53% | 0.85% |
| Tanzania | 8,085 | 0.074409 | 13,153,222,656 | 91.28 | 0.30% | 0.48% |
| Zambia | 2,845 | 0.081131 | 6,690,596,352 | 153.62 | 0.42% | 0.68% |
| Zimbabwe | 1,355 | 0.091164 | 6,030,266,368 | 524.74 | 0.63% | 1.01% |

**Supplemental Table 4: Deaths, life expectancy losses, and full income losses as a share of annual GDP, as a share of per capita GDP (2100).** Full income losses per person were derived by dividing the full income losses by the annual population projected for SSP2 by Samir et al. ^197^. Full income losses as a share of GDP per capita was derived by dividing the full income losses per person by the per capita GDP projected for SSP2 by Dellink et al. ^19^. Full income losses as a share of annual GDP was derived by dividing the annual full income losses by the annual GDP projected for SSP2 by Dellink et al. ^19^. All monetary values are reported in 2024 US$ rates.

*Sensitivity Analysis*

We conducted a probabilistic sensitivity analysis to evaluate the sensitivity of the full income estimates to all-cause mortality risks and economic parameter uncertainty and choice. We sampled 5000 draws for parameter values, from uniform distributions with parameter boundaries in Supplemental Tables 1 and 2, using a Latin Hypercube Sampling algorithm^15^. Sensitivity analysis was performed using Stata (IC version 14.2) and Microsoft Excel for Mac (version 16.45).

| ***Parameter*** | ***Upper and Lower Bound*** | ***Full income in 2025***  ***(2022 US$ billions)*** | ***Full income in 2050***  ***(2022 US$ billions)*** | ***Full income in 2100***  ***(2022 US$ billions)*** |
| --- | --- | --- | --- | --- |
| US VSL | $5,992,367 - $19,546,532 ^16^ | 3.32 – 10.84 | 12.96 – 42.29 | 102.72 – 335.08 |
| Income elasticity | 0.5 - 1.5 | 1.40 - 42.30 | 7.59 - 108.97 | 128.77 – 379.27 |
| Net all-cause mortality risk | 1.001 - 1.004^5^ | 2.96 – 11.87 | 11.61 – 46.30 | 92.66 – 366.28 |

**Supplemental Table 5: Sensitivity of full income estimates to key parameters values – For 2025, 2050 and 2100**

**
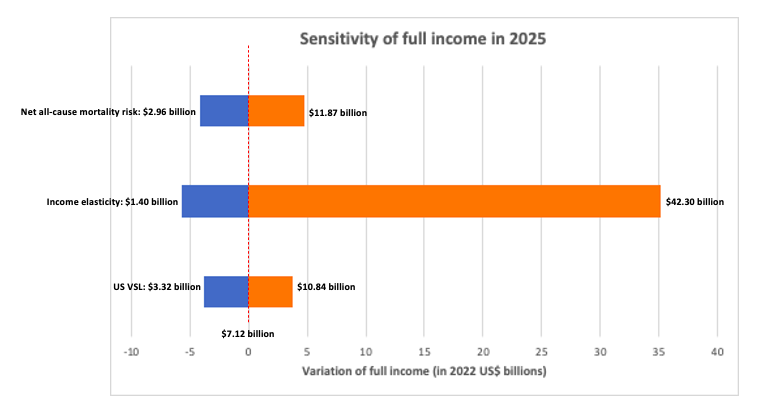
**

**Supplemental Figure 2: Sensitivity of full income estimates to parameter values (2025).** Tornado diagram of the sensitivity of full income estimates to bounds of all-cause mortality risk change for a unit change (1^0^C) in ambient temperature, bounds of income elasticity used in benefits transfer, and bounds of VSL.

**
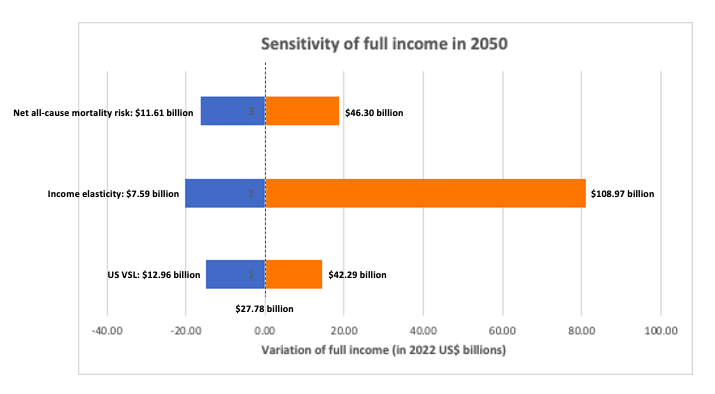
**

**Supplemental Figure 3: Sensitivity of full income estimates to parameter values (2050).** Tornado diagram of the sensitivity of full income estimates to bounds of all-cause mortality risk change for a unit change (1^0^C) in ambient temperature, bounds of income elasticity used in benefits transfer, and bounds of VSL.

**
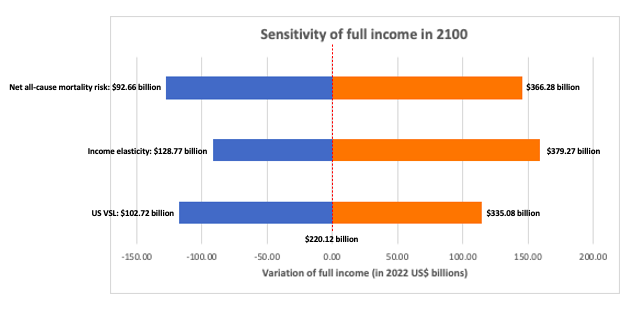
**

**Supplemental Figure 4: Sensitivity of full income estimates to parameter values (2100).** Tornado diagram of the sensitivity of full income estimates to bounds of all-cause mortality risk change for a unit change (1^0^C) in ambient temperature, bounds of income elasticity used in benefits transfer, and bounds of VSL.

We also used the empirically established value-of-a-statistical-life-year (VSLY) from Patenaude et al.^17^ to calculate full income losses in 2025, 2050 and 2100. To do so, we first converted the VSLY value and the median annual income for the 4000 respondents, from the reported 2015 international dollar (I$) value to 2022 US$. We then used the undiscounted life expectancy at the mean age of the respondents^18^ to convert the VSLY value to a VSL value in 2022 US$ rates. Using VSL and income values, we estimated the full income in 2025, 2050 and 2100.

| ***Parameter*** | ***Upper and Lower Bound*** | ***Full income in 2025***  ***(2022 US$ billion)*** | ***Full income in 2050***  ***(2022 US$ billion)*** | ***Full income in 2100***  ***(2022 US$ billion)*** |
| --- | --- | --- | --- | --- |
| VSL | $2,541,348^17^ | 10.22 | 39.84 | 315.21 |
| VSL Range | $1,688,609 - $3,366,606^17^ | 6.79 – 13.53 | 26.47 – 52.78 | 209.44 – 417.57 |

**Supplemental Table 6: Full income values calculated using empirically estimated VSLY values for Kenya.** In the calculations, the income elasticity was assumed to be 1.0^13^. The net all-cause mortality risk for sub-Saharan Africa was assumed to be 0.0024^6^

**References**

1. Meinshausen, M. *et al.* The shared socio-economic pathway (SSP) greenhouse gas concentrations and their extensions to 2500. *Geoscientific Model Development* **13**, 3571–3605 (2020).

2. Meinshausen, M., Raper, S. C. B. & Wigley, T. M. L. Emulating coupled atmosphere-ocean and carbon cycle models with a simpler model, MAGICC6 – Part 1: Model description and calibration. *Atmospheric Chemistry and Physics* **11**, 1417–1456 (2011).

3. Riahi, K. *et al.* The Shared Socioeconomic Pathways and their energy, land use, and greenhouse gas emissions implications: An overview. *Global Environmental Change* **42**, 153–168 (2017).

4. O’Neill, B. C. *et al.* The roads ahead: Narratives for shared socioeconomic pathways describing world futures in the 21st century. *Global Environmental Change* **42**, 169–180 (2017).

5. Cromar, K. R. *et al.* Global Health Impacts for Economic Models of Climate Change: A Systematic Review and Meta-Analysis. *Ann Am Thorac Soc* **19**, 1203–1212.

6. Cromar, K. R. *et al.* Global Health Impacts for Economic Models of Climate Change: A Systematic Review and Meta-Analysis. *Ann Am Thorac Soc* **19**, 1203–1212.

7. Kc, S. & Lutz, W. The human core of the shared socioeconomic pathways: Population scenarios by age, sex and level of education for all countries to 2100. *Glob Environ Change* **42**, 181–192 (2017).

8. World Population Prospects - Population Division - United Nations. https://population.un.org/wpp/.

9. World Population Prospects - Population Division - United Nations. https://esa.un.org/unpd/wpp/.

10. Preston, S., Heuveline, P. & Guillot, M. *Demography: Measuring and Modeling Population Processes*. (Wiley-Blackwell, Malden, MA, 2000).

11. Dellink, R., Chateau, J., Lanzi, E. & Magné, B. Long-term economic growth projections in the Shared Socioeconomic Pathways. *Global Environmental Change* **42**, 200–214 (2017).

12. U.S. Department of Health and Human Services. Appendix D: Updating Value per Statistical Life (VSL) Estimates for Inflation and Changes in Real Income. *ASPE* https://aspe.hhs.gov/reports/updating-vsl-estimates.

13. Hammitt, J. K. & Robinson, L. A. The Income Elasticity of the Value per Statistical Life: Transferring Estimates between High and Low Income Populations. *Journal of Benefit-Cost Analysis* **2**, 1–29 (2011).

14. African Development Bank. Climate Change in Africa. *African Development Bank Group* https://www.afdb.org/en/cop25/climate-change-africa (2019).

15. Orwa, T. O., Mbogo, R. W. & Luboobi, L. S. Uncertainty and Sensitivity Analysis Applied to an In-Host Malaria Model with Multiple Vaccine Antigens. *Int. J. Appl. Comput. Math* **5**, 73 (2019).

16. U.S. Department of Health and Human Services. Appendix D: Updating Value per Statistical Life (VSL) Estimates for Inflation and Changes in Real Income. *U.S. Department of Health and Human Services* https://aspe.hhs.gov/reports/updating-vsl-estimates (2021).

17. Patenaude, B. N., Semali, I., Killewo, J. & Bärnighausen, T. The Value of a Statistical Life-Year in Sub-Saharan Africa: Evidence From a Large Population-Based Survey in Tanzania. *Value Health Reg Issues* **19**, 151–156 (2019).

18. Kenya National Bureau of Statistics. Distribution of Kenya Population by Sex, Number of Households, Land Area, Population Density and County - Kenya Data Portal. *Knoema* https://kenya.opendataforafrica.org//fwjfdnc/distribution-of-kenya-population-by-sex-number-of-households-land-area-population-density-and-county.

19. K. C., S. *et al.* Updating the Shared Socioeconomic Pathways (SSPs) Global Population and Human Capital Projections. https://pure.iiasa.ac.at/id/eprint/19487/ (2024).
